# Health behaviors of blood donors in Saudi Arabia: a cross-sectional comparative study with non-donors

**DOI:** 10.3389/fpubh.2026.1944575

**Published:** 2026-09-23

**Authors:** Abdulrahman Alshalani, Hassan Abu Sabah, Ismael Sumili, Soha Moustafa, Yahia Alhammdi, Lamyaa A. Abu Ghabrah, Laila Otudi, Fahad Alshehri

**Affiliations:** 1 Department of Clinical Laboratory Sciences, College of Applied Medical Sciences, King Saud University, Riyadh, Saudi Arabia; 2 Haematology Division, Pathology and Clinical Laboratory Medicine Department, King Faisal Medical City for Southern Region, Abha, Saudi Arabia; 3 Laboratory and Blood Bank Department, Aseer Central Hospital, Aseer Health Cluster, Abha, Saudi Arabia

**Keywords:** blood donation, health promotion, lifestyle behaviors, physical activity, smoking cessation (SC)

## Abstract

**Background:**

Blood donation is essential for maintaining a safe and sustainable blood supply. Evidence suggests that blood donors may exhibit healthier lifestyle behaviors than non-donors (the healthy donor effect), but data from Saudi Arabia remain limited. This study compared lifestyle behaviors and health perceptions between blood donors and non-donors in Saudi Arabia.

**Methods:**

This cross-sectional study included 397 Saudi adults (265 blood donors and 132 non-donors) recruited between April and October 2024. Participants completed a structured questionnaire adapted from a previously published questionnaire assessing sociodemographic characteristics, lifestyle behaviors, and donation-related beliefs. Associations between donor status and selected lifestyle outcomes were examined using multivariable logistic regression adjusted for age, sex, BMI, education, occupation, and smoking where appropriate.

**Results:**

Compared with non-donors, donors were more likely to report regular or daily physical activity (adjusted odds ratio [aOR] 2.64, 95% CI 1.26–5.53), good or excellent meal quality (aOR 2.39, 95% CI 1.20–4.74), and good or excellent sleep quality (aOR 2.29, 95% CI 1.19–4.38). However, donors also had higher odds of regular smoking (aOR 2.81, 95% CI 1.16–6.79) and current smoking (aOR 3.62, 95% CI 1.69–7.75). Most donors (84.9%) believed that blood donation benefited their general health and activity.

**Conclusion:**

Blood donors reported more favorable behaviors related to physical activity, nutrition, and sleep than non-donors after adjustment for measured confounders. However, the higher prevalence of smoking among donors indicates that healthier behaviors do not extend uniformly across all lifestyle domains, and residual confounding related to baseline demographic differences cannot be excluded. Blood donation services may therefore provide valuable opportunities to integrate targeted health promotion and smoking cessation initiatives.

## Introduction

A safe, sufficient, and sustainable blood supply is an essential element of modern healthcare systems. Blood components are required for trauma care, surgery, hematological disorders, organ transplantation, and the management of many acute and chronic conditions. Because blood cannot be manufactured artificially at scale, health systems depend on the continuous participation of eligible donors. Voluntary blood donation is widely considered the most dependable model for maintaining transfusion safety and meeting demand for blood products (1, 2). However, maintaining an adequate donor pool remains a continuing challenge, particularly in settings where donation is replacement-based or strongly influenced by family and social networks.

Understanding the characteristics of people who donate blood is important for two related reasons. First, donor-related evidence helps blood services design effective recruitment, retention, and communication strategies. Second, blood donors represent a population that interacts with the healthcare system while generally feeling healthy, which creates an opportunity to link blood donation services with broader public health activities. Many previous studies have focused on donor demographics, motivations, and barriers to donation (3, 4). Comparatively less attention has been given to the broader lifestyle profile of donors, including their physical activity, dietary patterns, smoking status, sleep behavior, and health perceptions. These behaviors are particularly relevant because they may influence donor eligibility, donor well-being, and long-term retention.

A recurring observation in transfusion medicine and epidemiology is that blood donors often appear healthier than the general population. This phenomenon, commonly described as the healthy donor effect, reflects several overlapping mechanisms. Individuals who donate must meet eligibility criteria, including requirements related to general health, hemoglobin concentration, medical history, and infection risk. In addition, people who volunteer to donate may be more socially engaged, more health-aware, and more willing to participate in prosocial or preventive health behaviors. Consequently, donor populations may differ from non-donor populations in ways that are not solely attributable to the act of donation itself (5–7). International studies have reported that blood donors may be more likely to engage in physical activity, have favorable self-rated health, and avoid certain risk behaviors compared with non-donors (8–10). However, the healthy donor effect should not be treated as a universal or uniform pattern. Lifestyle behaviors are shaped by age, sex, education, employment, socioeconomic context, culture, religion, healthcare access, and local norms. Therefore, donor populations in different countries may show distinct behavioral profiles. Moreover, some behaviors may follow different directions: a donor group may be more active or nutritionally aware while still having a higher prevalence of smoking if the donor pool is predominantly male or older. For this reason, analyses comparing donors and non-donors should account for demographic differences whenever possible (11, 12).

The Saudi context is especially important for this research question. Saudi Arabia continues to face important public health challenges related to physical inactivity, unhealthy dietary patterns, obesity, sleep disruption, and tobacco use (13). These factors are relevant not only to chronic disease prevention but also to the sustainability of blood donation systems. At the same time, social, cultural, and religious values may strongly influence donation motives, including altruism, helping relatives, humanitarian responsibility, and religiously motivated giving (14). Despite this relevance, empirical evidence comparing donors and non-donors in Saudi Arabia remains limited.

This study aimed to compare lifestyle behaviors and health perceptions among blood donors and non-donors in Saudi Arabia. The specific objectives were to describe sociodemographic characteristics; examine differences in physical activity, dietary habits, smoking, and sleep patterns; evaluate donors’ perceived health benefits of donation; and perform adjusted analyses of selected lifestyle outcomes.

## Methods

### Study design and setting

This was a cross-sectional comparative study conducted in the Aseer region of southern Saudi Arabia between April and October 2024. Participants were recruited using convenience sampling during the same study period. Blood donors were recruited exclusively from the Donation Center at Aseer Central Hospital and were required to meet local blood donation eligibility criteria. Non-donors were Saudi adults who had never previously donated blood and were recruited from community-based settings in the Aseer region, including hospital visitor areas, and public areas. Potential non-donors were approached directly at these locations and invited to participate in the study. The donor and non-donor groups were not individually matched or frequency-matched on demographic characteristics. Non-Saudi participants and individuals who did not meet the study eligibility criteria were excluded. Ethical approval for this study was obtained from the Aseer Institutional Review Board (Aseer IRB), under approval number REC 7-4-2024.

The initial sample-size calculation was performed to determine the minimum number of blood donors required for recruitment and was based on the annual number of blood donors attending the Blood Bank Department of Aseer Central Hospital, using a 95% confidence level and a 5% margin of error. This yielded a minimum target of approximately 200 donors, which was exceeded by the final recruitment of 265 donors. An additional 132 non-donors were recruited as the comparison group, resulting in a total analytical sample of 397 participants. Because the original calculation was designed primarily to estimate the required donor sample rather than to detect a prespecified between-group difference in a particular lifestyle outcome, it did not incorporate an anticipated effect size, statistical power, donor-to-non-donor ratio, or expected non-response rate. This represents a limitation of the original sample-size planning and should be considered when interpreting the comparative analyses.

### Sample collection procedure

Data were collected using a structured questionnaire adapted from a previously published questionnaire (10). The questionnaire was modified to align with the objectives and local context of the present study. The questionnaire included three main sections. The first section captured sociodemographic and background characteristics, including sex, age, marital status, education level, employment status, occupation, smoking status, donation history, donation purpose, donation motive, weight, height, calculated body mass index, and residential region. The second section assessed lifestyle and health behaviors, including physical activity, nutritional habits, and sleep patterns. Physical activity items covered activity type, frequency, duration, intensity, location, and activity history. Nutrition items assessed the number of daily meals, perceived meal quality, and frequency of food consumption from restaurants. Sleep items assessed daily sleep duration, usual sleep timing, and perceived sleep quality. The third section focused on donor perceptions regarding the possible health, sleep, physical activity, and psychological benefits of blood donation.

### Outcome definitions and covariates

For descriptive analyses, questionnaire responses were retained and presented in their original categories. For the adjusted analyses, selected outcomes were grouped into binary categories to provide behaviorally meaningful and readily interpretable contrasts relevant to the study objectives. Physical activity frequency was classified as regular/daily versus rarely/occasionally, distinguishing participants reporting a consistent activity pattern from those reporting less consistent activity. Meal quality and sleep quality were classified as good/excellent versus poor/fair to distinguish favorable from less favorable self-reported patterns. Regular smoking was analyzed as a separate binary outcome, while current smoking was defined as occasional or regular smoking. These categorizations were based on the conceptual meaning of the questionnaire response categories rather than on data-derived statistical cut-points. Donor status was coded as donor versus non-donor, with non-donors used as the reference group. Covariates included age category, sex, BMI, education level, occupation, and smoking status where smoking was not the outcome.

### Statistical analysis

Descriptive statistics were used to summarize the study population. Categorical variables are presented as frequencies and percentages. Continuous BMI is presented as median and interquartile range (IQR) in the descriptive table. Group comparisons between donors and non-donors were performed using Pearson chi-square tests. A *p*-value less than 0.05 was considered statistically significant.

Multivariable binary logistic regression was used to examine the association between donor status and selected lifestyle outcomes. Donor status was the primary exposure variable, with non-donors used as the reference group. Covariates were specified *a priori* based on their potential confounding relationships with donor status and lifestyle behaviors and included age, sex, BMI, education level, occupation, and smoking status where smoking was not the outcome. All covariates were entered simultaneously into each model. Age was analyzed categorically because the questionnaire collected predefined age groups rather than exact age, whereas BMI was entered as a continuous variable. Sex, education, occupation, and smoking status were treated as categorical variables. Models examining regular/daily physical activity, good/excellent meal quality, and good/excellent sleep quality were adjusted for age, sex, BMI, education, occupation, and smoking status. Models examining regular smoking and current smoking were adjusted for age, sex, BMI, education, and occupation. Missing data were handled using complete-case analysis. Each adjusted model included 393 participants; the reduction from the overall sample of 397 reflects exclusion of observations with incomplete data for variables required in the respective regression model. Multicollinearity was assessed using variance inflation factors, model goodness-of-fit was evaluated using the Hosmer–Lemeshow test, and residual and influence diagnostics were examined to identify potentially influential observations. Results are presented as regression coefficients with standard errors, adjusted odds ratios, 95% confidence intervals, and *p*-values.

## Results

### Participant characteristics

The study included 397 participants, comprising 265 blood donors and 132 non-donors. The two groups differed substantially in several demographic characteristics (Table 1). Donors were overwhelmingly male, with males representing 98.5% of the donor group, whereas females represented 47.0% of the non-donor group. Donors also tended to be older, with the largest proportion in the 36-45-year category. Non-donors included a larger proportion of participants aged 18–25 years. Employment patterns differed between groups: donors were more frequently employed, whereas non-donors included more students and unemployed participants. Moreover, regular smoking was reported by 18.9% of donors compared with 6.8% of non-donors. Occasional smoking was also more common among donors. BMI was slightly higher among donors, with a median BMI of 27.65 kg/m2 compared with 26.69 kg/m2 among non-donors. Among donors, the most common stated purpose for blood donation was voluntary donation, followed by donation to help relatives or friends. The dominant motive for donation was helping patients, reported by 88.3% of donors.

**Table 1:** Demographic characteristics of the study participant.

Variable	Donor (*n* = 265)	Non-donor (*n* = 132)
Sex, *n* (%)		
Female	4 (1.5)	62 (47.0)
Male	261 (98.5)	70 (53.0)
Age, *n* (%)		
18–25	49 (18.5)	41 (31.1)
26–35	81 (30.6)	25 (18.9)
36–45	93 (35.1)	50 (37.9)
46–55	30 (11.3)	13 (9.8)
>55	12 (4.5)	3 (2.3)
Marital status, *n* (%)		
Single	92 (34.7)	51 (38.6)
Married	173 (65.3)	81 (61.4)
Education level, *n* (%)		
High School or Below	89 (33.6)	31 (23.5)
Diploma	23 (8.7)	0 (0.0)
BSc.	115 (43.4)	90 (68.2)
MSc.	30 (11.3)	9 (6.8)
PhD	8 (3.0)	2 (1.5)
Occupation, *n* (%)		
Self-employed	32 (12.1)	3 (2.3)
Employee	195 (73.6)	60 (45.5)
Student	11 (4.2)	35 (26.5)
Unemployed	27 (10.2)	34 (25.8)
Smoking, *n* (%)		
Non-Smoker	157 (59.2)	113 (85.6)
Former-Smoker	21 (7.9)	4 (3.0)
Occasional-Smoker	37 (14.0)	6 (4.5)
Regular-Smoker	50 (18.9)	9 (6.8)
**BMI, median (IQR)**	**27.65 (24.89–31.14)**	**26.69 (23.07–30.37)**
Purpose for your blood donation, *n* (%)		
Voluntary	103 (38.9)	
For relative help and friendship reasons	91 (34.3)	
For religious reasons	17 (6.4)	
For humanity and ethical reasons	39 (14.7)	
For medical benefits	15 (5.7)	
The Motive for your blood donation, *n* (%)		
To help patients	233 (88.3)	
To take a day off	4 (1.5)	
To get a donor prize	4 (1.5)	
Other	23 (8.7)	

BMI, Body mass index. Percentages for donation motive are based on available responses (*n* = 264).

### Physical activity patterns

Physical activity differed between donors and non-donors in several respects (Table 2). Walking was the most commonly reported physical activity in both groups and was more frequently reported by non-donors than donors (77.3% vs. 67.3%). In contrast, some activity types, particularly cycling, were more commonly reported among donors. Donors were more likely than non-donors to report regular or daily physical activity. Specifically, 17.0% of donors reported exercising regularly and 17.8% reported exercising daily, compared with 7.6 and 15.9% of non-donors, respectively. Physical activity duration also differed between groups, and donors were more likely to report activity lasting two hours or more. Activity location varied as well, with donors more frequently reporting use of public walking areas and gyms, while non-donors more often reported exercising at home.

**Table 2:** Lifestyle and health behaviors.

Variable	Donor (*n* = 265)	Non-donor (*n* = 132)	Pearson Chi-Square (*p*-value)
Physical activity types, *n* (%)			
Walking	177 (67.3)	102 (77.3)	12.70 (0.026)
Running	12 (4.6)	3 (2.3)	
Football	25 (9.5)	11 (8.3)	
Gymnastics	10 (3.8)	2 (1.5)	
Swimming	13 (4.9)	11 (8.3)	
Cycling	26 (9.9)	3 (2.3)	
Physical activity frequency, *n* (%)			
Rarely	40 (15.2)	27 (20.5)	7.95 (0.047)
Occasionally	132 (50.0)	74 (56.1)	
Regularly	45 (17.0)	10 (7.6)	
Daily	47 (17.8)	21 (15.9)	
Physical activity duration, *n* (%)			
15 min	50 (18.9)	29 (22.0)	9.67 (0.046)
30 min	100 (37.7)	39 (29.5)	
45 min	38 (14.3)	22 (16.7)	
1 h	44 (16.6)	34 (25.8)	
2 h or more	33 (12.5)	8 (6.1)	
Physical activity intensity, *n* (%)			
Low	61 (23.1)	44 (33.3)	4.74 (0.094)
Moderate	179 (67.8)	78 (59.1)	
High	24 (9.1)	10 (7.6)	
Physical activity location, *n* (%)			
Gyms/fitness center	50 (18.9)	20 (15.3)	15.73 (0.001)
Public walking area	146 (55.3)	64 (48.9)	
In house gym	27 (10.2)	33 (25.2)	
Other	41 (15.5)	14 (10.7)	
Physical activity history, *n* (%)			
Less than a year	100 (38.0)	59 (44.7)	1.64 (0.441)
Around a year	39 (14.8)	17 (12.9)	
More than a year	124 (47.1)	56 (42.4)	
Blood donation makes me physically active, *n* (%)			
Strongly agree	17 (6.4)		
Agree	29 (10.9)		
Neutral	78 (29.4)		
Disagree	81 (30.6)		
Strongly disagree	60 (22.6)		

### Nutrition behaviors

Dietary patterns also differed between donors and non-donors (Table 3). Donors were more likely to report eating three or more meals per day, whereas non-donors more frequently reported eating only one to two meals daily. Self-rated meal quality was higher among donors: 49.1% of donors rated their meals as good and 25.7% as excellent, compared with 38.6 and 15.2% of non-donors, respectively. The frequency of food consumption from restaurants did not differ meaningfully between groups, suggesting that the observed nutritional differences were more strongly related to meal frequency and perceived meal quality than to restaurant food consumption.

**Table 3:** Nutrition style.

Variable	Donor (*n* = 265)	Non-donor (*n* = 132)	Pearson Chi-Square (*p*-value)
Number of daily meals, *n* (%)			
1–2	86 (32.5)	65 (49.2)	10.81 (0.005)
3	157 (59.2)	57 (43.2)	
≥ 4	22 (8.3)	10 (7.6)	
Quality of meals, *n* (%)			
Poor	9 (3.4)	8 (6.1)	18.46 (<0.001)
Fair	58 (21.9)	53 (40.2)	
Good	130 (49.1)	51 (38.6)	
Excellent	68 (25.7)	20 (15.2)	
Food consumption from restaurants per month, *n* (%)			
Never	32 (12.1)	11 (8.3)	1.96 (0.581)
1–5 times	139 (52.5)	75 (56.8)	
6–10 times	57 (21.5)	25 (18.9)	
> 10 times	37 (14.0)	21 (15.9)	
The healthy food is basic support for blood donation, *n* (%)			
Strongly agree	22 (8.3)		
Agree	33 (12.5)		
Neutral	45 (17.0)		
Disagree	90 (34.0)		
Strongly disagree	75 (28.3)		

### Sleep patterns

Sleep duration, sleep timing, and sleep quality were assessed in both groups (Table 4). Most participants in both groups reported sleeping between six and nine hours per day. The proportion reporting less than six hours of sleep was higher among non-donors than donors, although the overall distribution of sleep hours did not differ significantly in the descriptive comparison. Sleep timing was similar between groups, with many participants reporting either primarily night-time sleep or a mixed pattern between night and morning sleep. Sleep quality showed a more favorable pattern among donors, with 47.2% reporting good sleep and 16.6% reporting excellent sleep, compared with 39.7 and 14.5% among non-donors.

**Table 4:** Sleep style.

Variable	Donor (*n* = 265)	Non-donor (*n* = 132)	Pearson Chi-Square (*p*-value)
Daily sleep hours, *n* (%)			
< 6 h	68 (25.7)	43 (32.6)	2.10 (0.349)
6–9 h	183 (69.1)	83 (62.9)	
> 9 h	14 (5.3)	6 (4.5)	
Daily sleep time, *n* (%)			
Mostly in the night time	126 (47.5)	64 (48.5)	3.48 (0.175)
Mostly in the morning time	11 (4.2)	1 (0.8)	
Mix between night and morning time	128 (48.3)	67 (50.8)	
Quality of sleep, *n* (%)			
Poor	16 (6.0)	16 (12.2)	5.80 (0.122)
Fair	80 (30.2)	44 (33.6)	
Good	125 (47.2)	52 (39.7)	
Excellent	44 (16.6)	19 (14.5)	
The adequate sleep is essential for the blood donation process, *n* (%)			
Strongly agree	85 (32.1)		
Agree	115 (43.4)		
Neutral	52 (19.6)		
Disagree	5 (1.9)		
Strongly disagree	8 (3.0)		

### Perceived health benefits of blood donation

Table 5 summarizes donors’ views on the health benefits of blood donation. A total of 84.9% of donors agreed or strongly agreed that blood donation benefited their general health and activity. In addition, 66.8% agreed or strongly agreed that regular blood donation improved their sleep status, and 76.6% agreed or strongly agreed that they felt relaxed and mentally refreshed after donating blood. These responses show that many donors view blood donation not only as a prosocial act but also as an experience associated with personal well-being.

**Table 5:** Health benefits.

Variable	Donor (*n* = 265)
Blood donation has a benefit on my general health and activity, *n* (%)	
Strongly agree	88 (33.2)
Agree	137 (51.7)
Neutral	39 (14.7)
Disagree	1 (0.4)
Strongly disagree	0 (0.0)
The regular blood donation improves my sleep status, *n* (%)	
Strongly agree	66 (24.9)
Agree	111 (41.9)
Neutral	77 (29.1)
Disagree	8 (3.0)
Strongly disagree	3 (1.1)
After blood donation, I feel relaxed and mind refreshed, *n* (%)	
Strongly agree	72 (27.2)
Agree	131 (49.4)
Neutral	56 (21.1)
Disagree	5 (1.9)
Strongly disagree	1 (0.4)

### Logistic regression models between donor status and selected lifestyle outcomes

Each adjusted regression model included 393 participants following complete-case analysis, compared with 397 participants in the overall study sample. In multivariable logistic regression (Table 6), the association between donor status and regular or daily physical activity remained statistically significant. Donors had 2.64 times the odds of reporting regular or daily physical activity compared with non-donors (95% CI 1.26–5.53; *p* = 0.010). Similarly, donor status remained associated with higher odds of reporting good or excellent meal quality. Donors had 2.39 times the adjusted odds of reporting good or excellent meal quality compared with non-donors (95% CI 1.20–4.74; *p* = 0.013). Moreover, donors had higher odds of reporting good or excellent sleep quality than non-donors (aOR 2.29, 95% CI 1.19–4.38; *p* = 0.013). Finally, donors had significantly higher odds of regular smoking than non-donors (aOR 2.81, 95% CI 1.16–6.79; *p* = 0.022). When current smoking was defined more broadly as either occasional or regular smoking, donors also had higher adjusted odds of current smoking (aOR 3.62, 95% CI 1.69–7.75; *p* < 0.001). These findings demonstrate that smoking prevention and cessation counseling could be integrated into donor-facing public health activities.

**Table 6:** Crude and adjusted associations between donor status and selected lifestyle outcomes.

Outcome	Crude OR	95% CI	B (SE)	Adjusted OR	95% CI	*p*-value
Regular/daily physical activity	1.74	1.08–2.80	0.971 (0.377)	2.64	1.26–5.53	0.010
Good/excellent meal quality	2.54	1.63–3.94	0.871 (0.350)	2.39	1.20–4.74	0.013
Good/excellent sleep quality	1.49	0.97–2.28	0.829 (0.332)	2.29	1.19–4.38	0.013
Regular smoking	3.18	1.51–6.68	1.033 (0.451)	2.81	1.16–6.79	0.022
Current smoking (occasional or regular)	3.81	2.10–6.92	1.286 (0.389)	3.62	1.69–7.75	<0.001

Reference group for donor status: non-donors. Crude odds ratios represent unadjusted associations between donor status and each outcome. Adjusted models for physical activity, meal quality, and sleep quality included age, sex, BMI, education level, occupation, and smoking status. Adjusted models for regular smoking and current smoking included age, sex, BMI, education level, and occupation. Adjusted analyses were based on complete cases (*n* = 393).

## Discussion

This study compared lifestyle behaviors and health perceptions between blood donors and non-donors in the Aseer region of Saudi Arabia. Donors reported more favorable patterns in physical activity, meal quality, and perceived sleep quality, while also showing a higher prevalence of smoking. These associations remained evident after adjustment for major measured confounders. However, the substantial baseline differences between donors and non-donors, particularly in sex, age, and employment status, limit the comparability of the two groups and mean that residual confounding cannot be excluded. The findings therefore suggest a heterogeneous lifestyle profile among blood donors rather than a uniformly healthier pattern, highlighting both potentially favorable behaviors and important health-risk behaviors within the donor population.

The overall pattern of higher physical activity among donors is consistent with the healthy donor effect described in previous literature (5–8). Individuals who donate blood may be more likely to consider themselves healthy, engage with health services, and participate in community-oriented activities. Donation eligibility criteria may also contribute to selection of healthier individuals. People with major illness, severe anemia, or recent medical problems may be deferred from donation, meaning that the donor pool is partly shaped by health-related selection. However, the present findings should not be interpreted as evidence that blood donation causes people to become more physically active. It is more plausible that physically active individuals are more likely to meet eligibility requirements, feel confident about donating, or identify with health-promoting behaviors.

The association between donor status and better self-rated meal quality also supports the idea that donors may be more health-aware. Donors were more likely to report three or more meals per day and to rate their meal quality as good or excellent. From a transfusion medicine perspective, nutrition is relevant because iron status, hydration, and general health influence donor eligibility and donor experience. Donors who understand the importance of nutrition may be more motivated to maintain adequate dietary habits, particularly if they have previously received donation-related advice. From a public health perspective, donation centers may therefore provide an opportunity to reinforce healthy nutrition messages, especially regarding iron-rich foods, balanced meals, hydration, and post-donation recovery.

The sleep findings should be interpreted carefully. Descriptively, donors had somewhat better perceived sleep quality, and the adjusted model suggested higher odds of good or excellent sleep quality among donors. However, sleep duration and timing did not show strong differences in the descriptive analysis. It is possible that donors who are generally more health-conscious are also more likely to maintain better sleep routines or perceive their sleep positively. It is also possible that differences in work schedules, age, and sex contribute to perceived sleep quality in ways not fully captured by the model. Because the outcome was self-reported sleep quality rather than objective sleep measurement, the result should be understood as a perception-based association.

A particularly important finding is the higher prevalence of smoking among donors. This result contrasts with the expectation that donors consistently show lower risk behaviors than non-donors. It also contrasts with some international studies in which donor populations demonstrate lower smoking prevalence (8–10). Several explanations are possible. First, the donor group in this study was overwhelmingly male, and smoking prevalence is often higher among men than women in many settings. Second, donors were more likely to be employed and older, both of which may be associated with different smoking patterns. Third, local social and occupational contexts may influence smoking behavior among men who donate blood. Although the association remained after adjustment for several measured covariates, residual confounding related to demographic differences between the groups cannot be excluded. Nevertheless, the higher smoking prevalence observed among donors warrants further investigation and may be relevant to future donor health initiatives.

Donor motivations in this study were strongly prosocial. Most donors reported that their primary motive was helping patients, and common stated purposes included voluntary donation and helping relatives or friends. These findings are consistent with previous research showing that altruism, benevolence, social responsibility, and community obligation are major drivers of donation behavior (3, 4, 15, 16). In Saudi Arabia, these motives may interact with religious and cultural values that emphasize helping others and supporting patients in need. Recruitment campaigns that combine altruistic messaging with culturally appropriate narratives may therefore be particularly effective.

The high proportion of donors who believed that donation benefits their general health, improves sleep, or helps them feel relaxed and mentally refreshed deserves careful interpretation. Positive perceptions may support donor satisfaction and repeat donation, but they may also reflect misconceptions or socially desirable responses. Donation services should provide balanced information: blood donation is safe for eligible individuals and has major societal benefits, but donors should not be told that donation is a proven treatment for improving health, sleep, or activity unless supported by robust evidence. Clear communication is especially important to maintain public trust and ethical recruitment practices.

The marked sex imbalance among donors is another important finding. Women represented only 1.5% of the donor group but nearly half of the non-donor group. Although this substantial imbalance limits the extent to which statistical adjustment can account for sex-related differences, the very low representation of women among donors is consistent with the broader blood-donation pattern reported in Saudi Arabia. Nationwide Ministry of Health data showed that females accounted for only 2.5% of 342,460 blood donations recorded at governmental donation centers in 2020 (17). Other Saudi studies have similarly documented marked female underrepresentation and identified several contributing factors, including anemia and low hemoglobin, uncertainty regarding eligibility, concerns about donation-related adverse effects, cultural misconceptions, and limited accessibility to donation services (18–20). Thus, the observed sex distribution is not unique to the present sample but appears to reflect an important structural characteristic of the Saudi donor population. Nevertheless, because only four female donors were included in our study, the adjusted analyses cannot reliably estimate donor-associated differences separately among men and women, and residual confounding by sex cannot be excluded. Future studies should therefore specifically recruit larger numbers of female donors and consider sex-stratified analyses.

The age distribution also has implications for donor sustainability. The largest donor proportion was among adults aged 36–45 years, while younger adults were more represented among non-donors. A donor pool concentrated among middle-aged adults may be stable in the short term but could create future recruitment challenges if younger adults are not effectively engaged. Many blood services target schools, universities, and workplaces because these settings provide access to first-time donors and allow donor habits to form early. The present findings suggest that additional strategies may be needed to engage younger Saudi adults in regular voluntary donation, while also retaining existing donors.

Several limitations should also be acknowledged. First, the cross-sectional design prevents causal inference. The study cannot determine whether healthier lifestyles increase the likelihood of donating blood, whether donation encourages healthier behaviors, or whether both are influenced by unmeasured factors such as health awareness, social engagement, or access to healthcare. Second, lifestyle behaviors were self-reported and may be affected by recall bias or social desirability bias. Third, donors and non-donors differed substantially in sex, age, and employment status, resulting in limited comparability between the groups for some baseline characteristics. Although these variables were included in the multivariable models, statistical adjustment cannot fully compensate for limited overlap between the donor and non-donor populations, and residual confounding remains possible. This limitation was particularly pronounced for sex, as only four donors (1.5%) were female compared with 62 (47.0%) non-donors, precluding meaningful sex-stratified analyses. In addition, the original sample-size calculation was based on estimating the required donor sample and was not specifically powered for a prespecified between-group difference in the lifestyle outcomes examined. Fourth, the study was conducted in a single region and may not be generalizable to all Saudi populations. Finally, some variables required categorization for analysis, which may reduce information and obscure more detailed dose–response patterns.

## Conclusion

Blood donors in this Saudi cohort were more likely than non-donors to report favorable lifestyle behaviors related to physical activity, nutrition, and sleep quality. These associations remained evident in adjusted analyses, suggesting a healthy donor pattern in selected behavioral domains. However, substantial baseline differences between donors and non-donors, particularly in sex, age, and employment status, limit direct comparability between the groups and leave the possibility of residual confounding. Notably, donors also had higher odds of smoking, demonstrating that donor populations are not uniformly healthier across all risk behaviors. The findings highlight the potential role of blood donation centers as platforms for health promotion, particularly for reinforcing healthy lifestyles and addressing tobacco use. Because of the cross-sectional design, self-reported measures, and demographic differences between groups, the findings should be interpreted as associations rather than causal effects. Broader, longitudinal, multicenter studies with better demographic balance between donor and non-donor groups are needed to confirm these findings and inform donor recruitment and public health strategies in Saudi Arabia.

## Data Availability

The raw data supporting the conclusions of this article will be made available by the authors, without undue reservation.
